# Factors Affecting Vitamin C Status and Prevalence of Deficiency: A Global Health Perspective

**DOI:** 10.3390/nu12071963

**Published:** 2020-07-01

**Authors:** Anitra C. Carr, Sam Rowe

**Affiliations:** 1 Nutrition in Medicine Research Group, Department of Pathology & Biomedical Science, University of Otago, Christchurch 8011, New Zealand; 2 Department of Clinical Sciences, Liverpool School of Tropical Medicine, Liverpool L35QA, UK; sam.rowe@lstmed.ac.uk

**Keywords:** vitamin C, vitamin C status, vitamin C deficiency, global health, dietary intake, obesity, smoking, communicable disease, infection, noncommunicable disease

## Abstract

A recent review of global vitamin C status has indicated a high prevalence of deficiency, particularly in low- and middle-income countries, as well as in specific subgroups within high-income countries. Here, we provide a narrative review of potential factors influencing vitamin C status globally. The in vivo status of vitamin C is primarily affected by dietary intake and supplement use, with those who supplement having a higher mean status and a lower prevalence of deficiency. Dietary intake can be influenced by cultural aspects such as traditional cooking practices and staple foods, with many staple foods, such as grains, contributing negligible vitamin C to the diet. Environmental factors can also affect vitamin C intake and status; these include geographic region, season, and climate, as well as pollution, the latter partly due to enhanced oxidative stress. Demographic factors such as sex, age, and race are known to affect vitamin C status, as do socioeconomic factors such as deprivation, education and social class, and institutionalization. Various health aspects can affect vitamin C status; these include body weight, pregnancy and lactation, genetic variants, smoking, and disease states, including severe infections as well as various noncommunicable diseases such as cardiovascular disease and cancer. Some of these factors have changed over time; therefore, we also explore if vitamin C status has shown temporal changes. Overall, there are numerous factors that can affect vitamin C status to different extents in various regions of the world. Many of these factors are not taken into consideration during the setting of global dietary intake recommendations for vitamin C.

## 1. Introduction

Due to random genetic mutations that have occurred during our evolution, humans have lost the ability to synthesize ascorbate in our livers [1]. Therefore, instead of being a normal liver metabolite, as is the case for most other animal species, ascorbate has instead become a vitamin and must be obtained through the diet to prevent hypovitaminosis C and outright deficiency [2]. In a recent review, we described the global status of vitamin C and the prevalence of deficiency as assessed by plasma concentrations [3]. Whilst vitamin C concentrations are normally tightly controlled in humans with adequate intake, a number of studies suggest that hypovitaminosis C and deficiency are not uncommon worldwide and may be very common in some settings. Vitamin C is well absorbed in small quantities. Uptake occurs primarily through the sodium-dependent vitamin C transporter-1 (SVCT-1), which is rapidly saturated, allowing relatively limited absorption of vitamin C per serving [4]. Vitamin C is then accumulated at varying concentrations in different body tissues [5]. The vitamin is excreted unchanged in the urine, but reuptake occurs in the renal tubules [6]. Further loss of vitamin C occurs through the oxidation of ascorbic acid to dehydroascorbic acid which may then be recycled to ascorbic acid or undergo further oxidative decomposition [7]. Thus, in vivo, vitamin C concentrations are affected by a range of factors that include dietary intake, absorption, distribution, oxidative decomposition, and elimination. 

Vitamin C acts as an important antioxidant and plays a myriad of functions in optimal health and prevention of disease [8,9]. Even mild insufficiency or hypovitaminosis C can be associated with symptoms such as low mood [10]. More severe deficiency can be associated with a range of clinical presentations [11]. Prolonged severe deficiency results in the clinical syndrome of scurvy, a condition that continues to be diagnosed in individuals and occasionally in public health outbreaks to this day [12]; if left untreated, scurvy is a fatal disease. The optimum intake and concentrations of vitamin C, however, have been subject to debate and recommended values are not universal [13]. Due to a range of benefits, a number of international authorities have increased their dietary recommendations for vitamin C from those previously recommended on the basis of preventing scurvy [13]. Although these recommendations generally take into account variations in requirements based on age, sex, pregnancy and lactation, and sometimes smoking status, there are many other factors that affect vitamin C status that are not taken into consideration by these global authorities. In this review, we highlight the numerous factors that impact on vitamin C status and prevalence of deficiency globally.

## 2. Dietary Factors Determining Vitamin C Status

### 2.1. Dietary Intake

**Figure 1:**
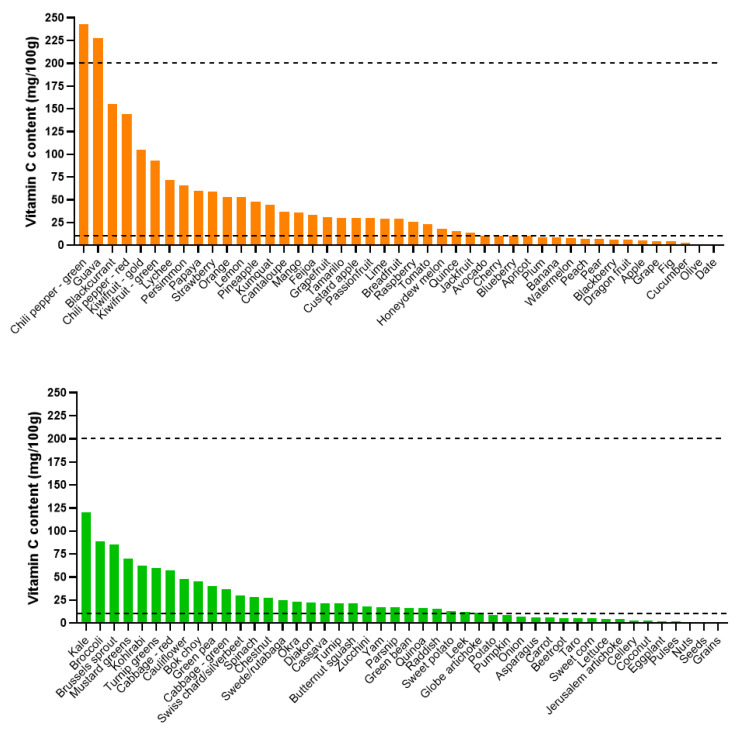
Estimated vitamin C content of selected fruit and vegetables. Data derived from the United States Department of Agriculture (https://fdc.nal.usda.gov/). Note that vitamin C content can vary depending on the plant variety, and cooking may decrease the vitamin C content to variable extents. Pulses include kidney beans, chickpeas, mung beans, pinto beans, soybeans, lentils, peanuts, split peas; nuts include hazelnuts, pistachios, macadamia nuts, pecans, walnuts, brazil nuts, cashew nuts; seeds include chia, flax seeds, pumpkin seeds, sunflower seeds, sesame seeds; grains include rice, millet, wheat/couscous, cornmeal. Animal products, such as meat (other than liver), eggs, and milk contain negligible vitamin C. Dotted lines: lower line indicates daily intake to prevent scurvy (10 mg/d); upper line indicates daily intake for optimal health (200 mg/d).

Dietary intake of vitamin C is a key determinant of body status, with the amount consumed and the frequency of consumption correlating with plasma status and prevalence of deficiency (Table 1) [14,15]. Fresh fruit and vegetables are the major dietary source of vitamin C, with fruit intake correlating more strongly with plasma vitamin C status [16,17]. Foods rich in vitamin C include oranges and other citrus fruit, guavas, kiwifruit, cranberries, strawberries, papayas, mangoes, melons, cantaloupe, spinach, Swiss chard, tomatoes, asparagus, and Brussels sprouts (Figure 1) [18]. In contrast, grains (e.g., rice, millet, wheat/couscous, corn), some starchy roots and tubers, meat (other than liver), eggs, and dairy contain very little vitamin C. 

Suboptimal diet is an important preventable risk factor for noncommunicable diseases, and a low intake of fruit was found to be the third leading dietary risk for deaths (two million deaths) and disability-adjusted life-years (65 million DALYs) globally [31]. The World Health Organization recommends at least 400 g (i.e., five portions) of fruit and vegetables per day, excluding potatoes, sweet potatoes, cassava, and other starchy roots [32]. Historically, scurvy has been reported in refugee camps in Ethiopia; the relief food distributed to the refugees was almost completely deficient in vitamin C, and the environment where the camps were located precluded an adequate supply of fresh food [33]. However, vitamin C deficiency may even be common in fertile areas of Africa, such as Uganda [34]. There have been a number of outbreaks of clinical scurvy in recent years, including in tribesmen in Kenya and during the Afghanistan winter [35,36], and cases of clinical scurvy continue to be diagnosed related to poor intake even in high-income countries [12,37]. 

### 2.2. Cultural Aspects: Staple Foods and Traditional Cooking Practices

Food consumption is influenced by a number of factors, such as geographical, economic, social, and cultural [25]. It is well known that economic factors, such as poverty, can limit the consumption of healthy foods. Furthermore, tradition and social customs may influence the consumption of a variety of foods. For example, a study in Nepal highlighted cultural beliefs around menstruation, pregnancy, and lactation that decreased micronutrient intake and intrahousehold disfavouritism towards women in the distribution of micronutrient-rich foods [27]. In low- and middle-income countries (LMICs), geographic considerations and agricultural practices influence the production and consumption of staple foods, which can greatly influence vitamin C intake. For example, countries whose populations consume grains such as rice and millet as staple foods (e.g., in Asia and parts of Africa) tend to have lower intakes of vitamin C [18,25]. In contrast, some areas of Africa and Latin America where yams and sweet potato are staple foods, vitamin C intakes may be higher. However, the vitamin C content of food can also vary depending on the time of harvest, transportation and storage conditions, and food preparation conditions. For example, the vitamin C content of some staple foods, e.g., cassava, is significantly depleted by different processing methods, which could lead to inaccuracies in estimated vitamin C intake [38]. Vitamin C is heat-labile and can be destroyed by cooking; the lower vitamin C status of Indians and Malays living in Singapore is thought to be partly due to its destruction by more prolonged cooking [26]. 

### 2.3. Supplement Use

An optimal vitamin C intake (i.e., 200 mg/d) can be obtained from five-plus daily servings of fresh fruit and vegetables, providing that at least one or two servings are high vitamin C foods, however, this is not always possible for many people around the world. Therefore, taking supplements in addition to dietary intake can help to maintain optimal vitamin C status. There are many different forms of vitamin C supplements; however, research indicates that these generally have the same bioavailability as food-derived vitamin C [39]. Numerous studies have found that even in developed countries, where there is no shortage of fresh fruit and vegetables, those who consume supplements have significantly higher vitamin C status and/or lower prevalence of deficiency (Table 2). Two large health surveys carried out in Canada (CHMS) and the USA (NHANES IV) reported 20% supplement use in Canada and 37 to 47% supplement use, for men and women, respectively, in the USA [14,15]. It should be noted, however, that in these surveys, supplement use was considered to be as little as once in the previous month. Nevertheless, both studies found vitamin C status was at least 20 µmol/L higher in supplement users, with a low prevalence of deficiency (0–2%) in those who supplemented [14,15]. Two studies in the UK indicated that people who did not supplement had a two-fold odds ratio of having a vitamin C status of <28 µmol/L and a three-fold odds ratio of their vitamin C status being <11 µmol/L, relative to concentrations >28 µmol/L [19,28].

## 3. Environmental Factors Affecting Vitamin C Status

### 3.1. Geographic Region

Geographic differences in vitamin C status have been reported (Table 3); there are likely many factors that contribute to the observed differences. For example, significant differences in vitamin C status and prevalence of deficiency were observed between Finland and neighboring Russia (where strikingly low plasma concentrations were observed) [17]. Geographical differences in the consumption of fresh fruit and vegetables were apparent, which were associated with deficiency. Low educational status was also noted to be associated with deficiency on the Russian side of the border [17]. Another study carried out in five countries across Europe indicated a difference in the vitamin C status of women, with the biggest differences being observed between Northern Ireland and the Republic of Ireland [49]. No differences in vitamin C status were observed for men from these different countries, whilst women had higher vitamin C status than the men in three of the five countries. Mosdol et al. [19] reported that low-income participants living in Scotland and Northern Island had a lower prevalence of deficiency than those living in England, whilst those living in Wales had a higher prevalence of deficiency. As part of the SU.VI.MAX trial, France was divided into seven regions, and vitamin C status was found to be significantly lower in the Northern region [50]. The reasons for these regional differences are unknown. In a large study of elderly people in India, a higher prevalence of deficiency was observed in the north (74% deficiency) compared with the south (46% deficiency) [24]. Similarly, in Mexico, a higher prevalence of deficiency was observed in children in the north and south regions (~26% deficiency) compared with those living in Mexico City (12% deficiency) [40]. This could reflect differences in the socioeconomic status between urban and rural environments.

### 3.2. Season and Climate

Since fresh fruit and vegetables are the major source of vitamin C for most people, it is perhaps not surprising that there have been reports of seasonal differences in vitamin C status (Table 3). Surprisingly, however, studies carried out in England and China have indicated that vitamin C status tends to be highest in winter and lowest in autumn, with up to 10 µmol/L difference between these seasons [30,52]. Another study in China indicated a much higher vitamin C status in winter relative to spring [53]. This likely reflects the types or amounts of vitamin C-rich foods being consumed in winter. Similar trends were observed in northern India, with less deficiency being observed in the winter months; however, in southern India, the winter months were associated with a higher prevalence of deficiency [24]. This likely reflects the different climatic and agricultural patterns across the subcontinent. It is possible that seasonal variances in plasma vitamin C concentrations are affected by baseline vitamin C status, i.e., whether the individual is already saturated or not. Bates et al. [59] reported seasonal variations in participants with higher intakes and blood status at baseline compared with little variation in those with low intakes and status, the latter likely due to a depleted body pool and preferential uptake of the vitamin by tissues. Changes in climate, such as drought, are also likely to impact on the vitamin C status of the populations reliant on local foods in the affected region [36]. This also leads to increased reliance on staple crops like cassava that lose their vitamin content prior to consumption [38]. 

### 3.3. Pollution

Currently, over half of the world’s population lives in urban areas, and WHO data indicate that more than 80% of people living in urban areas are exposed to air quality levels that exceed WHO guideline limits, with LMICs suffering from the highest exposures [60,61]. Air pollution causes an estimated seven million premature deaths worldwide every year, primarily resulting from increased mortality from strokes, heart disease, chronic obstructive pulmonary disease, lung cancer, and acute respiratory infections [61]. Environmental air pollution, such as smoke derived from burning biomass, can comprise reactive species that potentially affect in vivo antioxidant status [24]. Another example is environmental tobacco smoke, which is an underestimated pollutant in many parts of the world. Exposure to environmental tobacco smoke is associated with depleted vitamin C status in both nonsmoking adults and children [42,54,55,56,57,58]. Tribble et al. [54] found that the vitamin C status of passive smokers was significantly lower than that of nonexposed nonsmokers, despite comparable dietary intakes of the vitamin. Hypovitaminosis C was observed in 12% of passive smokers, but not in nonexposed nonsmokers. Analysis of the data of children from the NHANES III and IV surveys revealed a dose–response relationship between levels of tobacco exposure and serum vitamin C concentrations [55,56]. Not all studies in adults, however, have shown effects of passive smoking on vitamin C status [62,63,64,65]. Nevertheless, supplementation with vitamin C and other antioxidants was found to decrease oxidative biomarkers in participants exposed to environmental tobacco smoke [62,66]. 

## 4. Effect of Demographic Factors on Vitamin C Status

### 4.1. Sex

In high-income settings, females appear to have higher vitamin C status and a lower prevalence of deficiency than males [3]. According to McCall et al. [28], UK males have a four-fold odds ratio of deficiency compared with females (Table 4). The difference in vitamin C status between males and females is thought to be partly a result of a volumetric dilution effect due to the higher fat-free mass of males [29]. There are also differences in dietary intakes between men and women, with women generally having comparable or higher intakes than men in high-income countries, although this difference is less apparent in some low-income settings [3]. It should also be noted that pregnancy and lactation typically lower women’s vitamin C status due to hemodilution and the needs of the developing fetus and growing infant (see below). Many countries have higher dietary recommendations for men (to take into account the differences in body mass) and for pregnant and lactating women [13].

### 4.2. Age

Some studies carried out in people aged >60 years (e.g., the French POLA study and the British National Diet and Nutrition Survey) have shown lower vitamin C status than other studies carried out in the same countries with younger age groups (e.g., the French SU.VI.MAX study and the European EPIC-Norfolk study); however, it is not ideal to compare values between different studies due to potential methodological differences. A number of studies have indicated that older age within the same study population is associated with an increased prevalence of vitamin C deficiency, particularly in men, and older men also tend to have a lower vitamin C intake than older women [20,24,28,71]. However, a counterpoint to this is that overall vitamin C status can be higher in older people [15,29,50], although not in all cases, as was found in men in Finland who had lower vitamin C status with older age [72]. Schleicher et al. found a U-shaped curve for vitamin C status over the age range of 6 to 60+ years [15]. It is possible that the lower body mass of children and elderly, due to age-related frailty, could contribute to their higher vitamin C status. However, there are, clearly, subpopulations within the aging population who have increased deficiency, likely due to lower intake and/or comorbidities.

### 4.3. Race

A number of studies have indicated that vitamin C status varies by race (Table 4; reviewed in [74]). The prevalence of vitamin C deficiency appeared to be highest amongst South Asians and was thought to be partly due to traditional cooking practices. It was also suggested that the high proportion of individuals with low vitamin C concentrations in South Asian populations might contribute to their higher rates of cardiovascular disease. Only a few studies have assessed different ethnic groups within the same study [15,26,30,73]. In 1980, Koh et al. [73] assessed vitamin C status in a cohort of black and white participants in Mississippi, USA. Black males and females had significantly lower vitamin C status than white males and females (up to 10 µmol/L difference for females). The more recent US NHANES IV survey also found significantly lower vitamin C status in black females compared with white females [15]. In London, the vitamin C status of black and South Asians was significantly lower than white participants (up to 15 µmol/L lower for South Asian females) [30]. Similarly, in Singapore, Malays and Asian Indians were found to have significantly lower vitamin C status than Chinese (up to 12 µmol/L for females) [26]. Studies in Uganda, South Africa, and Nigeria all showed high rates of deficiency in predominately black Africans [3]. 

## 5. Effect of Socioeconomic Factors on Vitamin C Status

### 5.1. Socioeconomic Status/Deprivation

Socioeconomic status affects diet quality as foods of higher quality and higher nutritional value generally cost more [75]. Therefore, it is not surprising that numerous studies have shown that lower socioeconomic status and deprivation negatively impact on vitamin C status (Table 2). In a UK study, McCall et al. [28] reported that those who were most deprived (based on the Townsend deprivation index) had a two-fold odds ratio of vitamin C deficiency relative to those with plasma values above 28 µmol/L. This finding was supported by Mosdol et al. [19], who specifically focused on the low-income population within the UK, and showed a higher prevalence of deficiency and insufficiency than has been reported for the general UK population. Bates et al. [41] reported a three-fold difference in plasma vitamin C status for people of low socioeconomic status in the UK relative to those of higher status. A two-fold difference in vitamin C intake between the two socioeconomic groups likely contributed to the observed differences in vitamin C status. Other more recent surveys have confirmed the association of low vitamin C status with low socioeconomic status [15,43]. The British National Diet and Nutrition Survey indicated a 2 µmol/L increase in vitamin C status in adults for every additional £10,000 of income [44].

### 5.2. Education and Social Class

Similar findings have been reported for the level of education, with the lowest level of education associated with the lowest vitamin C status [14,17]. McCall et al. [28] reported that those with the lowest level of education in the UK had a 4.5-fold odds ratio of deficiency relative to a vitamin C status of >28 µmol/L. The same authors investigated social class and found that those having manual occupations had a three-fold odds ratio of deficiency compared with those having nonmanual occupations [28]. Similar findings were reported by Wreiden et al. [20], with two- to three-fold higher prevalence of vitamin C deficiency for females and males, respectively, with manual occupations. 

### 5.3. Institutionalization

One survey of elderly people assessed institutionalized individuals relative to free-living individuals [41]. The authors found significantly lower vitamin C status (25 vs. 44 µmol/L) and a higher prevalence of deficiency (40% vs. 14%) in institutionalized elderly compared with free-living elderly, respectively. Much of this is likely due to a higher proportion of individuals consuming less than the recommended nutrient intake for vitamin C in institutional settings [41]. This will become a growing concern with an increasingly aging population. Other institutionalized individuals, e.g., priests, prisoners, and boarding school children, have also been found to have lower vitamin C status, and higher hypovitaminosis C and severe deficiency, compared to control groups [46,47,48].

## 6. Health Aspects that Affect Vitamin C Status

### 6.1. Body Weight and Body Mass Index (BMI)

Bodyweight and the related BMI, waist circumference, or waist-to-hip ratio are well known to have a significant association with vitamin C status (Table 5). Numerous studies have shown an inverse association between body weight or BMI and vitamin C status, with obese individuals having the lowest vitamin C status [14,15,16,29,30,67,76]. Schleicher et al. [15] reported up to a 15 µmol/L difference in the vitamin C status of obese women compared with women of healthy weight (i.e., 45 versus 60 µmol/L, respectively). Pearson et al. [43] found that individuals with hypovitaminosis C (i.e., <23 µmol/L) had significantly higher weight, BMI, and waist circumference. Lower vitamin C status in those of the highest weight could be partly due to lower dietary intake of the vitamin [76,77]. In support of this, lower vitamin C status was found in those with the highest fat intake [23]. It has also been suggested that the observed differences in vitamin C status could be a result of volumetric dilution due to differences in fat-free mass [29]. This premise is supported by supplementation studies that have indicated that individuals with higher body weight do not replete as readily as those of normal body weight [78,79]. 

Based on these observations, Block et al. [78] proposed that recommended vitamin C intakes should be based on a dose per kg body weight or in terms of an ideal plasma concentration. This is a prudent recommendation considering the ongoing increase in body weight globally, with the prevalence of obesity exceeding 50% in some countries [80,81]. Obesity is a risk factor for numerous diseases, particularly cardiometabolic diseases, such as diabetes and cardiovascular disease, which are also associated with lower vitamin C status and a higher prevalence of deficiency [9]. Enhanced dietary fat and sugar, which are risk factors for cardiometabolic diseases, are also associated with decreased vitamin C intake and status [22,23].

### 6.2. Pregnancy and Lactation

Pregnant women typically have lower vitamin C status compared with nonpregnant women [34]. This is most likely due to hemodilution, as well as active transfer of the vitamin to the developing fetus [95]. Women with complications of pregnancy can have even lower vitamin C status [34,96]. Supplementation of pregnant women with vitamin C can potentially decrease the risk of some pregnancy-related complications [97]. Studies in high-income countries, which included women with normal vitamin C status, have failed to show benefit from supplementation [98]. However, studies in low-income settings with high rates of vitamin deficiency show potential benefits, including decreased rates of low birth weight, decreased hospital admissions, and possible decreased rates of pre-eclampsia [99]. It is noteworthy that the recently discovered epigenetic regulatory activities of vitamin C could have important roles to play in fetal development [100]. An animal model has indicated that maternal vitamin C can regulate the reprogramming of DNA methylation and germline development [101]. It is likely that lactating women also have lower vitamin C status due to the transfer of vitamin C to the growing infant via breastmilk. Many global authorities have taken the enhanced requirements of pregnant and lactating women into consideration, with recommendations above their standard dietary recommendations of +10–20 mg/d for pregnant women and +20–60 mg/d for lactating women [13].

### 6.3. Genetic Variants

Vitamin C status can potentially be influenced by genetic variants. The SLC23A1 gene encodes the sodium-dependent vitamin C transporter-1 (SVCT1), which is responsible for active uptake of dietary vitamin C through the intestinal epithelium and reuptake of filtered vitamin C via the kidney tubules and is essential in vitamin C-requiring animals [4,6]. A number of single nucleotide variants have been identified in the SLC23A1 gene, and modeling of in vitro data for four of these variants indicates 40% to 75% decreases in vitamin C uptake [6,102]. These variants are relatively common in those of African descent (6–16%), and the variant with the largest decrease in vitamin C transport has a high prevalence in African Americans [6]. Meta-analysis has indicated a strong association of another of these variants with decreased vitamin C status in five cohorts in the UK (frequency of ~3%) [45]. In the British Women’s Heart and Health Study, a further two variants, with relatively common frequencies of ~30%, were also found to be associated with vitamin C status [45]. Cahill et al. [84] reported that variants of SVCT1 can also modify the strength of the correlation between dietary vitamin C and serum vitamin C.

Vitamin C status could be further influenced by genetic variants affecting the metabolism of the vitamin [103]. A common variant of the hemoglobin-binding protein haptoglobin (Hp2-2) has a decreased ability to bind hemoglobin and results in increased oxidation of vitamin C in vitro [85]. Several studies have shown that this variant is associated with lower circulating vitamin C concentrations [85,86,87]. The Hp2-2 variant appeared to have a greater effect on individuals with dietary vitamin C intakes <90 mg/day [84]. It is noteworthy that genetic variants can show marked geographical differences; e.g., Hp2-2 is present in ~36% Northwest Europeans, 51% Iranians, 55% Thais and Chinese, and 84% Indians (where it is thought to have originated) [104]. Other genetic variants, such as those of the detoxifying enzyme glutathione S-transferase, may also have associations with vitamin C status, particularly in individuals with lower intakes, thereby resulting in an increased risk of deficiency [105]. High-dose vitamin supplementation has been shown to ameliorate certain gene variant defects [106] and is hypothesized to occur with vitamin C-related variants [107]. Therefore, individuals with genetic variants affecting vitamin C status may require higher dietary intakes. 

### 6.4. Smoking

Smoking is a well-known source of oxidants and in vivo oxidative stress [108]. Multiple studies have shown depleted vitamin C status and a higher prevalence of deficiency in smokers compared with nonsmokers (Table 5). McCall et al. [28] reported that current smokers had over a seven-fold odds ratio of deficiency compared with nonsmokers, and Wrieden et al. [20] showed two- to three-fold more deficiency in male and female smokers, respectively. Smokers tended to have dietary intakes that were lower in vitamin C, e.g., lower fruit and vegetable intake and higher fat intake [88,89,109,110]. However, when differences in dietary intake were taken into account, smokers still exhibited lower vitamin C status and higher requirements than nonsmokers [88,89,111,112]. Kallner et al. [113] demonstrated over a 40% increase in vitamin C turnover in smokers compared with nonsmokers. 

A few international regulatory authorities have taken the enhanced requirements of smokers into consideration with additional intake recommendations of 20 to 80 mg/d above their normal adult recommendations [13]. However, it is likely that these additional intakes are insufficient to compensate for the enhanced requirements of smokers [90]. Furthermore, some countries continue to show an increasing trend in smoking rates, which could potentially impact their population’s vitamin C status and requirements [114]. Of note, smoking cessation results in an increase in vitamin C status [115].

### 6.5. Disease States

Vitamin C status can be depleted by various disease states due to inflammatory processes and enhanced oxidative stress (reviewed in [9,92,93]). A number of studies of hospitalized patients showed a high prevalence of depleted plasma vitamin C status, and concentrations were inversely correlated with inflammatory markers [116,117,118,119]. A wide range of medical conditions are associated with vitamin C insufficiency; these include noncommunicable diseases such as cardiovascular diseases (e.g., strokes, coronary artery disease and hypertension), congestive heart failure, malignancy, chronic inflammatory states (e.g., rheumatoid arthritis), metabolic disorders (e.g., diabetes), and cataracts [9,92,93]. Acute infectious diseases leading to enhanced inflammation are also associated with depleted plasma vitamin C concentrations in plasma and immune cells, as are a range of chronic infections such as HIV, *Helicobacter pylori*, and tuberculosis, which are prevalent in many LMICs [94,120]. It should also be noted that requirements for vitamin C during infections increase with the severity of the infection, requiring significantly enhanced intakes to reach normal plasma status [94]. This is particularly pertinent to the current global coronavirus (SARS-CoV-2) pandemic, which causes severe pneumonia and sepsis, conditions known to be associated with significantly depleted vitamin C status [121,122]. It should also be noted that individuals with marginal vitamin C status are at higher risk of developing vitamin C deficiency [123], and once depleted, higher than recommended intakes of the vitamin are required to fully replete them again [78,79]. 

## 7. Has Vitamin C Status Changed Over Time? 

Various factors have changed over time that could potentially affect vitamin C status. These include population demographics and health status, e.g., obesity and smoking rates. Public health policies, such as recommended dietary intakes for vitamin C, have changed in many countries [13]. Therefore, have these temporal changes been reflected in changing vitamin C status over time? There have been relatively few studies that have assessed vitamin C status in the same populations or regions over time. The findings can also be complicated by changes in analytical methodologies over time. Two surveys have been carried out 19 years apart on elderly people in Britain: one in 1979 by the Department of Health and Social Security, and the National Diet and Nutrition Survey in 1998 [41]. There was an increase in vitamin C status from 29 to 44 µmol/L between the two surveys. However, it should be noted that participants in the first survey were older and analytical methodologies may have varied between the two surveys. In contrast, the more recent National Diet and Nutrition Surveys (2008–2017) have shown a significant 6% increase in vitamin C deficiency over the nine-year period in women aged 19–64 [44].

Schleicher et al. [15] carried out an indepth comparison of US vitamin C status in NHANES IV (2003–2004) relative to NHANES III that was undertaken 10–15 years earlier (i.e., 1988–1994) [124]. For those aged ≥20 years, overall vitamin C deficiency decreased by about 44% (from 15% to 8%) between the two surveys. Of note, there was an increase in the recommended dietary intake for vitamin C in the US in 2000, from 60 to 90 mg/d for men and to 75 mg/d for women [125]. However, it is unlikely that this increase in dietary recommendations fully accounts for the decrease in vitamin C deficiency over time as there were also some differences in methodology between the two surveys, with the more recent survey likely being more accurate [15]. Looking back even earlier to the NHANES II survey (carried out between 1976 and 1980), vitamin C status was reported to be higher for both men and women (i.e., ≥50 µmol/L for men and ≥60 µmol/L for women), with 5% deficiency reported, despite lower dietary intakes compared to the later NHANES III survey [126]. It should be noted, however, that vitamin C in the NHANES II samples was analyzed using the 2,4-dinitrophenylhydrazine method, which can be prone to interference by other components in plasma and thus potentially overestimate vitamin C concentrations [127].

Other time course data is available from studies carried out in Finland and neighboring Russia between 1992 and 2002 [17,51]. In North Karelia, Finland, the vitamin C status of men decreased from 54 to 27 µmol/L over 5 years, then rose slightly to 37 µmol/L over the next 5 year period [17]. Nyyssonen et al. reported a small increase in baseline vitamin C values in Finish men over 6 years [72]. In Pitkäranta, Russia, the vitamin C status of women decreased from 22 to 13 µmol/L over a 5-year period, while the vitamin C status of Russian men remained in the deficiency range over a 10-year period [17]. The blood samples from the more recent survey (2002) were from fasting participants; however, it is not clear whether they were fasting in the earlier surveys (1992 and 1997). 

Another set of studies was carried out in Java, Indonesia, between 2005 and 2011 [128,129]. Mean vitamin C status decreased in the subjects from 45 to 29 µmol/L over 6 years, although the percentage of deficiency decreased from 15% to 11% over this time. The decrease in mean vitamin C status could partly be explained by the earlier study assessing nonfasting plasma (as demonstrated by a higher percentage of vitamin C status >23 µmol/L, i.e., 72% versus 54%). However, there are possibly other environmental, lifestyle, or health-related issues that could be responsible for the changes in vitamin C status over time in Indonesia, Finland, and Russia.

Not all studies have shown changes in vitamin C status over time. A survey carried out in Shanghai, China, over a five-year period (between 1995 and 2000) showed no change in vitamin C status over this time period, although there was a dip in status at the midpoint (from 43 to 34 µmol/L) [52]. Thus, there is currently no clear trend in vitamin C status over time. However, much of the vitamin C data is very dated, and thus may not accurately reflect the current situation, particularly in countries where there have been changes over time in specific factors that can affect vitamin C status.

## 8. Conclusions and Future Directions

This narrative review describes the findings of numerous studies which highlight the various factors that can impact on vitamin C status and prevalence of deficiency. The studies were of variable quality; ideally, multivariate analysis of the various factors highlighted in these studies would be performed to further examine the relationships discussed. Another limitation is the variable quality of the processes and methodologies used to assess vitamin C status in the studies; this has been addressed in detail in our review on global vitamin C status [3].

Global vitamin C status appears to be a cause for concern, and we believe this is primarily due to poor diet, particularly in LMICs, although also apparent in subgroups within high-income settings [3]. Vitamin C concentrations provide a useful biomarker for a healthy diet; unfortunately, accurate analysis is costly, technically challenging, and time-consuming [130]. Thus, there is a need for low cost, accurate, and commercially available methods to assess plasma concentrations. Equally, the current assessment of intake is also limited and fails to take into account that the vitamin may be almost entirely eliminated prior to consumption in some globally important crops, such as cassava [38]. Future amendments to nutrient reference tables should take into this aspect into account. 

Clearly, there is a global need for increased consumption of vitamin-C-rich fresh fruit and vegetables. From our earlier review, this appears to be most notable in LMIC’s; however, it is also a concern in other at-risk groups [3]. Further education is required globally in how to meet the daily vitamin C requirements with locally available crops year-round. Some staple foods such as cassava require prolonged cooking to remove toxins (in this case, cyanide); efforts have been made to reduce this content in genetically modified cassava, which could allow higher vitamin C content from this vitamin-rich plant at the time of consumption [38]. Given the high rates of deficiency in some diets, cooking practices may also require modification to help increase intake. 

A number of studies have assessed the potential for vitamin C supplements to reduce a range of noncommunicable diseases and infections [9,94]. However, there remains much controversy as to the efficacy of supplementation, primarily due to badly designed studies that do not take into account the baseline vitamin C status of the participants [98]. Therefore, knowledge of the high-risk groups for deficiency should be utilized for future studies as these groups are more likely to benefit from supplementation. 

In the meantime, global and local policymakers should consider the local data available on deficiency in an attempt to modify dietary intake and other modifiable risk factors. More of these risk factors should be taken into account during the review and update of global recommended dietary intakes for the vitamin. Clinicians worldwide should also remain vigilant to detect and correct hypovitaminosis C and deficiency in the at-risk groups highlighted. 

## Figures and Tables

**Table 1:** Dietary factors determining vitamin C status.

Factor	Summary and Comments	References
Dietary intake	Dietary intake, particularly fruit intake, correlates with improved vitamin C status and decreased prevalence of deficiency; is dependent on the amount consumed, frequency of consumption, and type of food consumed as the vitamin C content of food varies. High dietary fat and sugar intake are associated with decreased vitamin C intake and status.	[14,15,16,17,19,20,21,22,23,24]
Staple foods	Staple foods such as grains (e.g., rice, millet, wheat/couscous, corn) and some starchy roots and tubers are low in vitamin C; populations who consume these staples can have lower overall vitamin C intake.	[18,25]
Traditional cooking practices	Through boiling or steaming, water-soluble vitamins may be leached from food and prolonged cooking of food can destroy vitamin C; this could lead to decreased vitamin C status in certain social or ethnic groups. Drying of leafy vegetables also decreases water-soluble vitamins.	[26,27]
Supplement use	Supplement users have significantly higher vitamin C status and negligible prevalence of deficiency. Non-users have a 2–3 fold odds ratio of insufficient and deficient vitamin C status.	[14,15,19,28,29,30]

**Table 2:** Effect of socioeconomic factors on vitamin C status.

Factor	Summary and Comments	References
Socioeconomic status/deprivation	Individuals with lower socioeconomic status or higher deprivation have lower vitamin C status and a higher prevalence of deficiency; this is partly due to the higher cost of good quality, nutrient-dense food.	[15,19,21,24,28,40,41,42,43,44,45]
Education and social class	Similarly, individuals with lower education and manual occupations have lower vitamin C status.	[14,17,20,28,42]
Institutionalized	Institutionalized elderly, and other institutionalized individuals (e.g., priests, prisoners, boarding school children) have lower vitamin C status and a higher prevalence of deficiency; this is partly due to a lower dietary intake.	[41,46,47,48]

**Table 3:** Environmental factors affecting vitamin C status.

Factor	Summary and Comments	References
Geographical region	Vitamin C status varies by geographical region, both within and between countries; this could partly reflect differences in socioeconomic status and available foods.	[17,19,24,40,41,49,50,51]
Season	Vitamin C status varies seasonally between countries, likely reflecting different crops and thus the types and/or amounts of vitamin C-rich foods consumed.	[24,30,52,53]
Climate	Drought and harsh winter climates have been associated with outbreaks of clinical scurvy.	[36]
Pollution	Exposure to environmental pollutants, e.g., smoke, can deplete vitamin C status; this is partly due to enhanced oxidative stress.	[24,42,54,55,56,57,58]

**Table 4:** Effect of demographic factors on vitamin C status.

Factor	Summary and Comments	References
Sex	Males generally have lower vitamin C status, and a higher prevalence of deficiency, than females; this is partly a result of a volumetric dilution effect due to the higher fat-free mass of males. This difference is less apparent in some low- and middle-income countries.	[14,15,17,19,22,24,26,28,29,30,42,43,49,50,53,67,68,69,70]
Age	Both children and elderly tend to have higher vitamin C status in high-income settings; this could partly be due to lower body weight. Elderly can have a higher prevalence of vitamin C deficiency in some settings; this could be due to lower intake and/or comorbidities.	[14,15,20,24,28,29,41,50,71,72]
Race	In the US and UK, African-Caribbean and South Asian people had a lower status than Caucasians. In South Asia, Malays and Indians had a lower status than Chinese; this is thought to be partly due to differences in culinary practices. Differences are more apparent between women of different races.	[15,26,30,73,74]

**Table 5:** Health aspects that affect vitamin C status.

Factor	Summary and Comments	References
Bodyweight, BMI	Individuals with higher body weight or BMI have lower vitamin C status; this is likely partly due to a volumetric dilution effect.	[14,15,16,24,29,30,43,67,76,77,82,83]
Physical activity	Physical activity level positively correlates with vitamin C status, with inactive individuals having a 3-fold odds ratio of deficiency; this is likely partly due to associated lifestyle factors such as diet and body weight.	[28,29]
Pregnancy and lactation	Pregnancy is associated with lower vitamin C status; this is partly due to hemodilution and active transfer of vitamin C to the developing fetus and growing infant via breastmilk.	[34]
Genetic variants	Polymorphisms in the genes for the vitamin C transporter (SVCT1) and haptoglobin (Hp2-2) are associated with lower vitamin C status; the latter is thought to be due to enhanced oxidative stress.	[6,45,84,85,86,87]
Smoking	Smokers have lower vitamin C status and a higher prevalence of deficiency than nonsmokers; this is partly due to enhanced oxidative stress.	[14,15,16,19,20,24,28,29,30,43,45,50,53,54,67,71,88,89,90,91]
Disease states	Various communicable and noncommunicable diseases are associated with lower vitamin C status; this is partly due to inflammatory processes and enhanced oxidative stress.	[9,92,93,94]
